# Associations of cardiorespiratory fitness, physical activity, and obesity with metabolic syndrome in Hong Kong Chinese midlife women

**DOI:** 10.1186/1471-2458-13-614

**Published:** 2013-06-27

**Authors:** Ruby Yu, Forrest Yau, Suzanne C Ho, Jean Woo

**Affiliations:** 1 Department of Medicine and Therapeutics, The Chinese University of Hong Kong, Shatin, Hong Kong, China; 2 School of Public Health and Primary Care, The Chinese University of Hong Kong, Shatin, Hong Kong, China

**Keywords:** Physical activity, Cardiorespiratory fitness, Chinese, Maximal oxygen consumption, Metabolic syndrome, Midlife women

## Abstract

**Background:**

Several studies have simultaneously examined physical activity (PA) and cardiorespiratory fitness (CRF) with metabolic syndrome (MS). However, the independent roles of both PA and CRF with MS are less firmly established. The combined contributions of PA and CRF with MS are less studied, particularly among Chinese women. There is uncertainty over the extent to which metabolically healthy but overweight/obese individuals have a higher CRF level.

**Methods:**

The sample included 184 Chinese women aged 55 to 69 years with available metabolic data and lifestyle factors. PA was assessed by self-reported questionnaire; CRF was assessed by maximal oxygen consumption (VO_2max_) during a symptom-limited maximal exercise test on a cycle ergometer. Metabolically healthy/abnormal was defined on the basis of absence or presence of MS. Overweight was defined as a body mass index (BMI) of ≥ 23 kg/m^2^ and obese was defined as a BMI of ≥ 25 kg/m^2^.

**Results:**

The prevalence of MS was 21.7%. PA was inversely associated with the prevalence of MS after adjustment for age, BMI, and dietary total calories intake, but the association was eliminated after further adjustment for CRF. CRF was inversely associated with the prevalence of MS independent of age, BMI, and dietary total calories intake, and the association remained significant after further adjustment for PA. In the PA and CRF combined analysis, compared with those in the lowest tertile of PA (inactive) and lowest tertile of CRF (unfit), the OR (95%CI) of having MS was 0.31 (0.09–1.06) for subjects in the higher tertiles (2^nd^–3^rd^) of PA (active) but were unfit, 0.23 (0.06–0.88) for subjects who were inactive but in the higher tertiles (2^nd^–3^rd^) of CRF (fit), and 0.14 (0.04–0.45) for subjects who were active and fit. Metabolically healthy but overweight/obese subjects had a higher CRF level than their metabolically abnormal and overweight/obese peers. However, the difference did not reach statistically significance.

**Conclusions:**

CRF has greater association with the prevalence of MS compared with PA in Chinese midlife women. The interrelationships between CRF, obesity, and MS needs further study.

## Background

Metabolic syndrome (MS), a clustering of three or more obesity-related risk factors namely high waist circumference, high triglycerides, high blood pressure, high blood glucose and low high-density lipoprotein (HDL) cholesterol [1], has emerged as an important risk factor for cardiovascular disease [2] and is associated with morbidity and all-cause mortality [3,4]. The prevalence of MS increases with age [5], and is highly prevalent among midlife women, with the rates varying from 23.2 to 35.1% [6-9]. The exact origin of this condition is less certain, but hormonal changes have been implicated as a causal factor for the increasing risk of MS at the menopausal transition [10,11]. Besides menopausal hormonal changes, interactions of genetic and behavioral factors also contribute to clustering of metabolic risk factors [12]. Therefore, clinical guidelines and strategies indicate that healthy eating and active lifestyle are the frontline approaches to preventing MS [1].

Substantial evidence demonstrates an inverse association of physical activity (PA) and cardiorespiratory fitness (CRF) with risk of MS in middle-aged and older populations [13-27]. The protective effects of higher levels of PA or CRF on MS are evident regardless of age, sex, body composition, smoking, alcohol intake and other clinical factors. PA is a behavior, defined as any bodily movement that increases energy expenditure, including both leisure time and non-leisure time activities, whereas CRF is a physiologic attribute, usually measured by a maximal or submaximal exercise test, and expressed as maximal oxygen uptake (VO_2max_). Compared with self-reported PA, CRF is a more accurate [28] and is thought to be stronger as a predictor of health outcomes because it is less prone to misclassification. Although CRF is partly determined by levels of PA, PA and CRF may be differentially influenced by body composition, environmental factors as well as genetic components [29]. Therefore the influence of PA and CRF on MS may occur through separate pathways.

Although several studies have simultaneously examined PA and CRF with MS [14,15,17,18,23,25], the independent roles of both PA and CRF with MS are less firmly established. The combined contributions of PA and CRF with MS are less studied. Although in a previous population-based study, middle-aged men with both sedentary lifestyle and poor CRF were associated with increased risk of MS [15], this study has been carried out among Caucasians, who may differ significantly from Chinese in terms of lifestyle, diet, and body physiology. For example, the age-defined VO_2max_ was noted to differ between Chinese adult men and women and their age-matched Caucasians adults [30]. Previously we have examined the normative values of CRF in Chinese midlife and elderly women. Although similar VO_2max_ values were observed as those of same sex and comparable age in Western populations, the VO_2max_ values being in the 5-15^th^ percentile values from the norms of the Cooper Institute [31,32].

There is evidence suggesting that higher CRF levels are associated with fewer metabolic complications and lower risk of heart disease or cancer across different weight status groups. Ortega et al. [33] have recently reported that metabolically healthy but obese middle-age individuals had better fitness than their metabolically abnormal obese peers, and for a given fitness level, the metabolically healthy but obese phenotype had a lower risk of all-cause mortality, non-fatal and fatal cardiovascular disease, and cancer mortality. The authors suggested that fitness assessment can contribute to properly define a subset of obese individuals who do not have an elevated risk of cardiovascular disease or cancer. However, the authors used the cut-off point of a BMI ≥ 30 kg/m^2^ for obesity which may not be suitable for Asian adults, who have different body build and body composition [34]. Moreover, the prevalence of MS in Chinese women is high [35]. Further research is needed to examine the relative and combined associations of PA and CRF with the risk of MS, and to understand whether higher CRF is a common feature in metabolically healthy but obese individuals, particularly among Chinese population.

The aim of the present study was to examine the cross-sectional relative and combined associations of PA and CRF with the risk of MS in a population-based sample of Hong Kong Chinese midlife women, taking into account for the potential confounding factors including age, BMI, and dietary total calories intake. We also aimed to test whether CRF is more highly associated with MS than PA, whether the associations between PA/CRF and MS are different across BMI categories, and whether metabolically healthy but obese individuals have higher CRF level.

## Methods

### Subjects

Five hundred and eighteen Hong Kong Chinese postmenopausal women aged 50 to 64 years were recruited for a study to examine the prevalence of subclinical atherosclerosis and its associated risk factors during 2002-2004. Subjects were recruited by random telephone dialing based on the most recent residential telephone directory. At least 6 attempts were made at different times of the day and week for each number before it was considered a noncontact. If more than one postmenopausal woman within the household fell into the targeted age range of 50 to 64 years, the member with the most recent birthday was selected. Women with surgical menopause, cardiovascular disease, and severe disease conditions such as cancer and renal failure were excluded. Eligible women were invited for questionnaire interviews, clinical examinations, and ultrasound measurements. A response rate of 62.5% was obtained. Details of the sampling method and of the baseline cohort have been reported elsewhere [36].

Between 2008 and 2009, the cohort was invited to re-attend for repeat questionnaire interviews, clinical examinations, and ultrasound measurements. The procedure for data collection was the same as baseline and follow-up. 414 of the 518 subjects (79.9%) returned. Of these individuals, those who were ambulant without assistance from another person were subsequently invited for the VO_2max_ assessment. Those who reported that they had leg pain, pace maker implanted, or were taking blood thinning medications were excluded. In addition, subjects who had systolic blood pressure > 199 mm Hg or diastolic blood pressure > 109 mm Hg, heart rate < 40 or > 110 beats per minute, had evidence of abnormal resting or exercise electrocardiogram, or had severe disease conditions such as heart disease, cancer, or renal failure were excluded. Hence, the present study population consisted of 184 subjects who had participated in the follow-up examinations with completed data on blood pressures, anthropometric and metabolic profiles, VO_2max_, and other confounding factors. Written informed consent was obtained from each subject and the study was approved by the Ethics Committees of the Chinese University of Hong Kong.

### Clinical examinations

Subjects fasted for 12 h before attending the study centre. Venous samples of blood were collected for the measurement of total cholesterol, HDL cholesterol, low-density lipoprotein (LDL) cholesterol, triglycerides, and fasting blood glucose. Blood samples were analyzed using commercial kits (Randox, United Kingdom) with standard enzymatic methods on the Alcyon 300 analyzer (Abbott Laboratories, Abbott Park, IL). Blood pressures were measured twice with a mercury sphygmomanometer on the right upper arm with the subject seated quietly for at least 10 minutes. Height, weight, waist, and hip circumferences were also measured twice with the subject wearing light clothing and no shoes. BMI was documented. Waist circumference was measured over the abdomen at the smallest diameter between the costal margin and iliac crest and hip circumference was measured at the level of the greater trochanters. Waist-to-hip ratio was calculated as the ratio of waist-to-hip circumferences.

According to the NCEP ATP III guidelines [1] and the revised cut-off value of waist circumference for defining abdominal obesity for Asian populations [37], a subject was classified as having MS if she had three or more of the following risk factors: 1) high blood pressure (systolic blood pressure ≥ 130 mm Hg and/or diastolic blood pressure ≥ 85 mm Hg and/or drug treatment), 2) abdominal obesity (waist circumference ≥ 80 cm), 3) high triglycerides (triglycerides ≥ 1.7 mmol/L), 4) low HDL (HDL < 1.3 mmol/L), and 5) high fasting blood glucose (fasting blood glucose ≥ 6.1 mmol/L and/or drug treatment).

### Assessment of physical activity

PA was assessed using the modified and locally translated Baecke questionnaire [38,39]. Based on the original Baecke questionnaire that was divided into three parts (work, sport and leisure time) [38], a section assessing activities on housework and additional cultural specific leisure time activities such as window shopping and playing mahjong (popular activities in Hong Kong) were included to make the questionnaire applicable for use in Hong Kong Chinese adults of a wide age range. The questions thus consisted of PA at work, in doing housework, at leisure time, and in doing sports. For each question, the subject had to choose subjectively from four choices: frequently, sometimes, rarely, or never. The scores from the four sections were used to derive the work index, housework index, leisure time index, and sport index. Summing the four indices result in a continuous unweighted total index. A weighted total index was also computed, taking into account the different time and activity pattern of workers and housewives. The reference period of the questionnaire refers to activities of the last year. Previously we found that the weighted total index was significantly correlated with daily energy expenditure obtained from the diaries and the total index was not [39]. However, the present results indicated that the distribution of weighted total index was skewed and the total index had a higher correlation with MS than the weighted total index. Therefore, total index were used as opposed to weighted total index in this study.

### Assessment of cardiorespiratory fitness

VO_2max_ was assessed with a symptom-limited maximal exercise test on an electrically braked bicycle ergometer (Ergoline 900, Ergoline GmbH, Lindenstrasse, Bitz, Germany). Subjects were instructed to abstain from any strenuous exercise on the day before testing. Each subject was connected to a calibrated respiratory gas analyzer (Fitmate, COSMED Srl, Italy), a portable metabolic analyzer designed for measurement of oxygen consumption during exercise via a face mask. It uses a turbine flowmeter for measuring ventilation and a galvanic fuel cell oxygen sensor for analyzing oxygen in expired gases, and a new sampling technology has been adopted through the use of a small sample of the expired volume in a miniaturized dynamic mixing chamber. Though it does not have a CO_2_ analyzer, it ramps the respiratory exchange ratio between 0.8 and 1.2 based on the increase in heart rate, thus markedly reduces to minimal error. As reported by Nieman and colleagues [40], there were no significant differences in oxygen consumption between the Fitmate systems and the Douglas bag system, the ‘gold standard’ for gas exchange measurements, during graded treadmill exercise. In the present study, the Fitmate analyzer was calibrated before each test. Blood pressure was monitored throughout the exercise test. The test started with a 3-min warm up at a workload of 20 W and continued with 10 W increments every minute, until the subject was exhausted or was not able to maintain the required pedaling frequency of 50 rpm. Subjects were verbally encouraged to reach their maximum. The test was terminated when the subject reached VO_2max_[41,42] or showed any symptoms that indicated termination of exercise based on the guidelines of the American College of Sports Medicine [43]. To check the test-retest reliability and the internal consistency of the assessment, a separate sample of 50 healthy men and 25 healthy women were invited to undergo a repeated VO_2max_ assessment within two weeks after the original assessment period. Paired sample t tests indicated that there were no significant mean differences between the two assessments (p = 0.051 for men and 0.054 for women). Internal consistency was also adequate with *Cronbach’s α* = 0.95 for men and 0.98 for women.

### Other covariates

Information on a number of covariates was also collected. Marital status, education level, occupation, medical history, current use of medications, smoking, and alcohol intake were obtained by questionnaire interview. A validated food frequency questionnaire containing 60 food items was used to assess dietary intake [44].

### Statistical analysis

Continuous variables are reported as mean and standard deviations, and categorical variables as percentages. Student t tests / Chi square tests were performed to compare subjects with MS and without MS. Analyses of Covariance (ANCOVA) were also performed after adjustment for age. To examine the relative association of PA and CRF with the risk of MS, PA and CRF were categorized into tertiles. The lowest tertile of PA (total index < 8.6) was designated as inactive and the higher tertiles (2^nd^–3^rd^) of PA (total index 8.6–< 9.4 and ≥ 9.4) were grouped together and designated as active. Similarly, the lowest tertile of CRF (VO_2max_ < 21.2 ml/kg/min) was designated as unfit and the higher tertiles (2^nd^–3^rd^) of CRF (VO_2max_ 21.2–< 24.3 and ≥ 24.3 ml/kg/min) were grouped together and designated as fit. The cut-off scores for the lowest terile of PA/CRF were used as the cut-off scores for inactive/active and unfit/fit because the results might provide a reference for the minimum PA/CRF level that is associated with lower risk of having MS. Logistic regression was used to estimate odds ratios (OR) and 95% confidence interval (CI) as an index of association of PA and CRF with prevalent MS. Models were adjusted for age, BMI and dietary total calories intake. In addition, PA was further adjusted in the models for CRF and CRF was further adjusted in the models for PA. The analyses were repeat stratified for PA, CRF, and BMI. The combined effects of PA and CRF on MS were also examined using logistic regression. For this analysis, four PA–CRF combination categories (inactive and unfit; inactive but fit; active but unfit; active and fit) were created where the effect of each combination of PA and CRF categories were compared with the referent group (inactive and unfit). To test whether metabolically healthy but obese subjects have a higher CRF level, subjects were categorized into four groups (metabolically healthy and normal weight, metabolically healthy but overweight/obese, metabolically unhealthy but normal weight, metabolically abnormal and overweight/obese) on the basis of absence or presence of MS and BMI levels of < 23 kg/m^2^ or ≥ 23 kg/m^2^. A cut-off point of a BMI of 23 kg/m^2^ was chosen because it represents the current standard for overweight, as proposed by the WHO Western Pacific Regional Office [37]. In this analysis, the waist circumference was excluded as a criterion in the definition of MS, since the purpose was to examine the CRF level across metabolic profile regardless of their adiposity. Comparisons of CRF level were made between the four categories using ANCOVA, with adjustment for age, dietary total calories intake, and PA. Pairwise comparisons were adjusted for the Bonferroni correction. All analyses were conducted with the Window-based SPSS Statistical Package (version 17.0; SPSS Inc., Chicago, IL), and P values less than 0.05 was considered statistically significant.

## Results

A total of 184 Chinese midlife women were examined (mean age, 61.1 ± 3.1 years; range, 54.9–69.4 years) The majority of the subjects were married (75.7%), worked as a housewife (71.7%), and had primary level of education (97.8%). The prevalence of MS was 21.7%. The mean PA score was 9.1 ± 1.4 (range 5.9 to 13.1) and the mean VO_2max_ was 22.8 ± 3.8 ml/kg/min (range 13.8 to 35.7 ml/kg/min). Subjects with MS had a significantly lower VO_2max_ that those without it (P < 0.0001) (Table 1).

**Table 1:** Characteristics of the study population according to the presence of metabolic syndrome

	**Mean ± SD / Frequency (%)**				
	**All**	**Without MS**	**With MS**		
**Characteristic**	**(n = 184)**	**(n = 144)**	**(n = 40)**	**P**	**P**^**1**^
Age, year	61.1 ± 3.1	61.2 ± 3.1	60.9 ± 3.1	0.680	—
Marital status, now married	139 (75.5)	108 (75.0)	31 (77.5)	0.745	—
Education attainment, primary or above	180 (97.8)	141 (97.9)	39 (97.5)	0.873	—
Occupation, housewife	132 (71.7)	102 (70.8)	30 (75.0)	0.605	—
Medical history					
Hypertension	37 (20.1)	18 (12.5)	19 (47.5)	0.000	—
Hypercholesterolemia	54 (30.5)	42 (30.2)	12 (31.6)	0.872	—
Diabetes	14 (7.7)	4 (2.8)	10 (25.0)	0.000	—
Use of medication					
Anti-hypertensive	34 (18.5)	16 (11.1)	18 (45.0)	0.000	—
Lipid-lowering	17 (9.2)	12 (8.3)	5 (12.5)	0.421	—
Anti-diabetic	13 (7.1)	3 (2.1)	10 (25.0)	0.000	—
Anthropometric and metabolic factor					
Systolic blood pressure, mmHg	120.6 ± 17.5	118.2 ± 15.7	128.3 ± 20.5	0.001	0.001
Diastolic blood pressure, mmHg	71.9 ± 8.1	71.5 ± 7.9	73.0 ± 8.1	0.296	0.316
BMI, kg/m^2^	23.4 ± 3.0	22.7 ± 2.6	26.1 ± 2.9	0.000	0.000
Waist circumference, cm	78.5 ± 7.9	76.4 ± 7.0	86.0 ± 6.7	0.000	0.000
Waist-hip-ratio	0.85 ± 0.05	0.84 ± 0.05	0.88 ± 0.04	0.000	0.000
Total cholesterol, mmol/L	5.3 ± 0.9	5.4 ± 0.9	5.0 ± 0.7	0.018	0.020
HDL cholesterol, mmol/L	1.6 ± 0.5	1.8 ± 0.4	1.2 ± 0.2	0.000	0.000
LDL cholesterol, mmol/L	3.1 ± 0.8	3.2 ± 0.9	2.9 ± 0.6	0.034	0.098
Triglycerides, mmol/L	1.3 ± 0.8	1.1 ± 0.4	2.2 ± 1.3	0.000	0.000
Fasting blood glucose, mmol/L	5.2 ± 0.9	5.0 ± 0.7	5.8 ± 1.1	0.000	0.000
Lifestyle factor					
Current smoker	2 (1.1)	1 (0.7)	1 (2.5)	0.330	—
Regular drinking*	9 (4.9)	6 (4.2)	3 (7.5)	0.387	—
Dietary total calories intake, kcal/day	1341.7 ± 400.9	1354.8 ± 380.8	1294.6 ± 468.7	0.403	0.377
PA, total index	9.1 ± 1.5	9.2 ± 1.5	8.9 ± 1.2	0.394	0.364
CRF, VO_2max_, ml/kg/min	22.8 ± 3.8	23.5 ± 3.4	20.1 ± 3.9	0.000	0.000

Data are presented as mean ± SD for continuous variables and number and percentage for categorical variables.

^1^Adjusted for age.

*Regular drinking was defined as reporting at least one drink per week.

*BMI* Body mass index, *CRF* Cardiorespiratory fitness, *HDL* High-density lipoprotein cholesterol, *LDL* Low-density lipoprotein cholesterol, *PA* Physical activity, *VO*_*2max*_ Maximal oxygen consumption, and *MS* Metabolic syndrome.

After adjustment for age, BMI, and dietary total calories intake, PA was inversely associated with the prevalence of MS (P = 0.04); however, the association was eliminated after further adjustment for CRF (P = 0.09). CRF was inversely associated with the prevalence of MS independent of age, BMI, and dietary total calories intake, and the association remained significant after further adjustment for PA (P = 0.01). The percentage contribution (R^2^) of PA with MS ranged from 0.043 to 0.403 while that of CRF ranged from 0.192 to 0.367 (Table 2).

**Table 2:** Prevalence and odds ratio within 95% confidence interval for metabolic syndrome across physical activity and cardiorespiratory fitness categories

			**Model 1**		**Model 2**		**Model 3**		**Model 4**	
	**No. (%) of MS**	**P**	**OR (95% CI)**	**P**	**OR (95% CI)**	**P**	**OR (95% CI)**	**P**	**OR (95% CI)**	**P**
PA										
Inactive	19 (31.7)	0.023	1.0 (reference)		1.0 (reference)		1.0 (reference)		1.0 (reference)	
Active	21 (16.9)		0.44 (0.21–0.90)	0.024	0.42 (0.18–0.97)	0.041	0.42 (0.18–0.98)	0.044	0.47 (0.20–1.13)	0.090
Nagelkerke R^2^			0.043		0.348		0.348		0.403	
CRF										
Unfit	26 (43.3)	0.000	1.0 (reference)		1.0 (reference)		1.0 (reference)		1.0 (reference)	
Fit	14 (11.3)		0.16 (0.07–0.34)	0.000	0.31 (0.13–0.73)	0.008	0.31 (0.13–0.74)	0.008	0.31 (0.13–0.76)	0.010
Nagelkerke R^2^			0.192		0.367		0.367		0.367	

Data are presented as number and percentage for categorical variables and OR (95% CI).

Model 1 adjusted for age (year).

Model 2 adjusted for age (year) and BMI (kg/m^2^).

Model 3 adjusted for age (year), BMI (kg/m^2^) and dietary total calories intake (kcal/day).

Model 4 adjusted for age (year), BMI (kg/m^2^), dietary total calories intake (kcal/day) and PA for CRF / CRF for PA.

*CRF* Cardiorespiratory fitness, *PA* Physical activity, and *MS* Metabolic syndrome.

In the CRF stratified analyses, PA was not associated with the prevalence of MS within both unfit and fit categories. On the other hand, in the PA stratified analyses, CRF was associated inversely with the prevalence of MS within subjects who were inactive (OR 0.18, 95%CI 1.05 to 0.66, P = 0.01). However, OR of having MS was not significant within subjects who were active (P = 0.38) (Table 3).

**Table 3:** Relative risk of having metabolic syndrome by physical activity in cardiorespiratory fitness stratified analysis and by cardiorespiratory fitness in physical activity stratified analysis

	**Unfit**			**Fit**		
	**No. (%) of MS**	**OR (95% CI)**	**P**	**No. (%) of MS**	**OR (95% CI)**	**P**
PA						
Inactive	14 (56.0)	1 (reference)		5 (14.3)	1 (reference)	
Active	12 (34.3)	0.68 (0.20–2.32)	0.543	9 (10.1)	0.28 (0.07–1.07)	0.062
	Inactive			Active		
	No. (%) of MS	OR (95% CI)	P	No. (%) of MS	OR (95% CI)	P
CRF						
Unfit	14 (56.0)	1 (reference)		12 (34.3)	1 (reference)	
Fit	5 (14.3)	0.18 (0.05–0.66)	0.010	9 (10.1)	0.55 (0.15–2.09)	0.381

Data are presented as number and percentage for categorical variables and OR (95% CI).

Models were adjusted for age (year), BMI (kg/m^2^) and dietary total calories intake (kcal/day).

CRF, cardiorespiratory fitness; PA, physical activity; and MS, metabolic syndrome.

To further examine whether adiposity confounds the associations of PA and CRF with MS, subsequent analyses were performed stratified by BMI whereas PA was associated inversely with the prevalence of MS within subjects who were normal weight (OR 0.12, 95%CI 0.04 to 0.39, P < 0.0001). However, OR of having MS was not significant within subjects who were obese (P = 0.17). High CRF had a beneficial effect on MS for those in the normal weight category (OR 0.26, 95%CI 0.09 to 0.77, P = 0.02). The corresponding OR was 0.16 (95%CI 0.04 to 0.59, P = 0.01) within those who were obese (Table 4).

**Table 4:** Relative risk of having metabolic syndrome by physical activity and cardiorespiratory fitness in BMI stratified analysis

	**BMI < 25 kg/m**^**2**^			**BMI ≥ 25 kg/m**^**2**^		
	**No. (%) of MS**	**OR (95% CI)**	**P**	**No. (%) of MS**	**OR (95% CI)**	**P**
PA						
Inactive	12 (29.3)	1 (reference)		7 (36.8)	1 (reference)	
Active	5 (5.5)	0.12 (0.04–0.39)	<0.0001	16 (48.5)	2.5 (0.67–9.64)	0.173
CRF						
Unfit	8 (25.0)	1 (reference)		18 (64.3)	1 (reference)	
Fit	9 (9.0)	0.26 (0.09–0.77)	0.015	5 (20.8)	0.16 (0.04–0.59)	0.006

Data are presented as number and percentage for categorical variables and OR (95% CI).

Models were adjusted for age (year) and dietary total calories intake (kcal/day).

*BMI* body mass index, *CRF* cardiorespiratory fitness, *PA* physical activity, and *MS* metabolic syndrome.

In the combined associations of PA and CRF with MS, subjects who were fit had significantly lower risk of having MS whether or not they were active (OR 0.14, 95%CI 0.04 to 0.45, P = 0.001) or inactive (OR 0.23, 95%CI 0.06 to 0.88, P = 0.03); however, if subjects were active but unfit, risk of having MS was not lower compared with the referent group that were classified as inactive and unfit (Table 5).

**Table 5:** Combined associations of physical activity and cardiorespiratory fitness with metabolic syndrome

	**Unfit**			**Fit**		
	**No. (%) of MS**	**OR (95% CI)**	**P**	**No. (%) of MS**	**OR (95% CI)**	**P**
PA						
Inactive	14 (56.0)	1 (reference)		5 (14.3)	0.23 (0.06–0.88)	0.031
Active	12 (34.3)	0.31 (0.09–1.06)	0.061	9 (10.1)	0.14 (0.04–0.45)	0.001

Data are presented as number and percentage for categorical variables and OR (95% CI).

Models were adjusted for age (year), BMI (kg/m^2^) and dietary total calories intake (kcal/day).

*PA* Physical activity and *MS* Metabolic syndrome.

Table 6 shows BMI and CRF levels across the four MS/BMI categories. After adjustment for age, dietary total calories intake, and PA, metabolically healthy but overweight/obese subjects had higher CRF than their metabolically abnormal and overweight/obese peers, but the difference did not reach statistically significance. A decreasing trend in the level of CRF was observed across the categories (P for trend <0.0001).

**Table 6:** Body mass index and cardiorespiratory fitness levels in metabolically healthy but overweight/obese subjects compared with metabolically healthy and normal weight, metabolically abnormal but normal weight, and metabolically abnormal and overweight/obese subjects

	**n**	**BMI, kg/m**^**2**^	**CRF (VO**_**2max**_**), ml/kg/min**	**P**	**P for trend**
Metabolically healthy and normal weight	83	20.8 ± 1.4	24.0 ± 3.6	0.000	0.000
Metabolically healthy but overweight/obese	84	25.4 ± 1.8	22.2 ± 3.4^*^		
Metabolically abnormal and normal weight	3	21.4 ± 1.1	20.1 ± 5.6		
Metabolically abnormal and overweight/obese	14	27.4 ± 2.4	19.6 ± 4.0^*^		

Data are presented as mean ± SD.

The model was adjusted for age (year), dietary total calories intake (kcal/day), and PA. Waist circumference was excluded as a criterion in the definition of MS.

*P < 0.01 when compared with the metabolically healthy and normal weight subjects. Pairwise comparisons were adjusted for the Bonferroni correction.

*BMI* Body mass index and *CRF* Cardiorespiratory fitness.

## Discussion

Results of the present study showed that PA was not associated as strongly as CRF with the prevalence of MS and the risk reduction was larger in subjects who were fit than those who were active after adjusting for age, BMI, and dietary total calories intake. Subjects who were active had 58% lower risk of having MS, but the association was no longer significant after adjustment for CRF. Subjects who were fit had 69% lower risk, and the association remained significant after further adjustment for PA. In stratified analyses, CRF was significantly associated with the risk of MS within inactive subjects. In combined analysis, subjects who were inactive but fit had lower risks of having MS. However, if subjects were active and unfit, the OR of having MS was not significantly lower than the referent group that was inactive and unfit.

Our findings are generally consistent with extensive research that has documented the inverse associations between PA and MS [14,15,17,18,23,25]. However, after adjustment for CRF, the association between PA and MS observed in our study became attenuated. In stratified analyses, no significant association between PA and the prevalence of MS was observed within unfit or fit categories. These findings must be interpreted with caution given the imprecise measurement associated with self-reported PA since self-reported data are more prone to recall bias and misclassification. Furthermore, the lack of statistical significance is likely explained by the small number of MS in fit subjects (n = 5). However, Laaksonen et al. [14] reported that middle-aged men without MS who complied with the PA recommendations had reduced risk of developing MS by about one-half compared with those engaging in no more than 60 minutes of moderate exercise per week, independent of CRF. The Medical Research Council (MRC) Ely Study showed that PA remained associated with MS and its progression after adjustment for CRF [17,18]. It has also been shown that increasing levels of PA may protect against MS even in the absence of improved CRF [23]. The disparate findings may be due to the use of different PA measurements, in that PA was measured objectively with individually calibrated heart rate against energy expenditure in the MRC Ely Study, which is more precise compared with self-report data; and this may partially explain the relatively stronger associations found between PA and MS than with CRF. However, in contrast to this notion, several studies have stated that leisure-time PA not resulting in an increase in CRF may not provide any protective effect on cardiovascular disease or its risk factors [45,46]. Results from the Aerobics center longitudinal study also demonstrated that the association of PA with all-cause mortality was eliminated after controlling for CRF [47]. Therefore, the independent role of PA on risk of MS is not confirmed. It is reasonable to suggest that the lower levels of CRF that are normally associated with PA are at least partially responsible for our findings.

Our results also agree with previous cross-sectional [24] and longitudinal studies [26] suggesting that low CRF is an independent risk factor of MS. Based on the baseline data of the Dose-Responses to Exercise Training Study (DR’s EXTRA), older men and women aged 57-79 years who were in the lowest tertile of VO_2max_ had a 10-fold higher risk of MS compared with those in the highest tertile [24]. Based on the baseline and 2-year follow-up data of the same study, those who were in the highest tertile of baseline VO_2max_ were 68% less likely to develop MS than those in the lowest tertile [26]. To check whether CRF contributes to the risk of MS independently of PA, PA was further adjusted and the association between CRF and MS remained significant, with subjects who were fit had 69% lower risk of MS. However, the MRC Ely Study showed contradictory results, with the association between CRF and MS attenuated after adjustment for objectively measured PA [17]. Therefore, whether the CRF effects on risk reduction for MS risk differ between PA levels is not firmly established.

The mechanisms by which moderate-to-high CRF provides a beneficial effect on the metabolic risk still needs to be determined but it is reasonable to believe that the benefit may be largely mediated by components of MS. A previous study in 297 apparently healthy men showed that the high CRF group had lower triglyceride levels and higher HDL cholesterol levels than the low-or moderate-CRF groups, independent of abdominal subcutaneous and visceral fat [20]. The finding of this study showing the independent association between CRF and MS for a given level of BMI lends further support to this observation.

Few studies have simultaneously examined PA and CRF on the risk of MS using combined stratification analysis, although one previous study in middle-aged men found that low levels of PA and CRF were associated with MS [15]. Since CRF is a strong correlate of PA, and the influence of PA and CRF on MS may occur through separate pathways, we examined the combined association of PA and CRF with the prevalence of MS, and similar associations were observed. Our results also showed that the combined effects of PA and CRF with MS were stronger than the single relative risks of having MS in fit subjects. However, although PA is an important determinant of CRF, genetic variation has a significant effect on response to exercise and thus CRF [29,48]. Recently, Timmons et al. [49] pointed out different individuals may respond differently to exercise and some individuals respond well to aerobic exercise with increased CRF while others did not. Therefore, incorporation of CRF into individual risk assessment may provide an efficient method for identifying individuals who would benefit from interventions to preventing MS.

In contrast with previous studies showing a higher CRF level among metabolically healthy but obese subjects than their metabolically abnormal and obese peers [33,50], we did not observe significant differences in level of CRF between the two groups in this study. Perhaps the small sample size of metabolically abnormal and overweight/obese subjects (n = 14) attenuated the statistical power. Differences in characteristics and the methods to identify obesity are also likely to contribute in part to the discrepancies between the studies. In the study of Ortega et al. [33] a mean BMI of 25.8 ± 4.0 kg/m^2^ was reported and the author defined obese as a BMI of ≥ 30 kg/m^2^ and compared the metabolically healthy and normal weight phenotype with the metabolically healthy but obese / metabolically abnormal and obese groups, leaving out those with overweight in the analyses. In a second study, Messier et al. [50] used dual-energy X-ray absorptiometry and computed tomography scan as methods to identify metabolically healthy but obese subjects. In contrast with these studies, the mean BMI was lower in our study (23.4 ± 3.0 kg/m^2^). Moreover, we defined overweight as a BMI of ≥ 23 kg/m^2^ and obese as a BMI of ≥ 25 kg/m^2^ and divided subjects into two groups, one group representing normal weight, and the other representing overweight/obese. Previous evidence suggested that findings from studies of Caucasians should not be extrapolated to other ethnic groups such as Asians from whom other cut-off points have been defined for obesity [51]. The Cooperative Meta-analysis group of the working group in obesity in China who suggest defining overweight as BMI ≥ 24 to 27.9 kg/m^2^ and obesity as BMI ≥ 28 kg/m^2^[52]. Nevertheless, there appears to be trend of a decreasing CRF levels across the MS/BMI categories, regardless of age, dietary total calories intake, and PA. Therefore, the findings of this study lend some support to the previous literature on the role of CRF on the risk of MS, and suggest that public health guidelines may need to be modified by placing more emphasis on the CRF level, especially for the midlife women.

Several studies have reported the prevalence of MS among midlife women, from 23.2 to 35.1% across different populations [6-9]. The prevalence in Chinese women is also high [35], in that people of Asian origin tend to accumulate more body fat and develop cardiovascular risk factors at lower BMI levels or smaller waist circumference than Caucasians [34]. However, the prevalence of MS in our study (21.7%) was lower than that reported from an earlier study in China. In the study of 181 postmenopausal women conducted in 2006-2008, the prevalence of MS was 33.7% [9]. Variation in the prevalence of MS could be due to heterogeneity of population characteristics such as age distribution, socioeconomic status or nutritional status, or due to different genetic background.

This study has several strengths, in that we were able to assess VO_2max_ directly by respiratory gas analysis during a maximum exercise test, and such analyses have been less studied in Chinese, particularly among midlife women. It is recognized that VO_2max_ is an accurate measure of CRF and an objective measure of recent patterns of PA, which is less prone to misclassification than self-reported PA, and this may partially explain the relatively weaker associations found between MS and PA than with CRF in this study. Other strengths of this study include the adjustment for multiple potential confounders, including dietary intake, which is known to influence the components of MS [27]. In a public health perspective, our findings have important implications, in highlighting that moderate-to-high CRF may reduce metabolic risk in midlife women, who represent a group of individuals at higher risk of future cardiovascular disease. Although PA may be less predictive of health outcomes compared with CRF, it is a primary modifiable factor to improve CRF despite some individuals may not respond well to aerobic exercise [48,49]. Therefore, health care providers should encourage their patients to become more fit by participating in regular PA to reduce MS risk.

There are some limitations in this study. The subjects were not representative of the Hong Kong population, in that their education level was higher. PA was self-reported such that the measurement accuracy is inferior to that of physical fitness in quality; therefore the difference in result between PA and CRF may be partly a reflection of this measurement accuracy. The sample size was small and the cross-sectional design also does not allow us to infer a causal relationship of PA and CRF with MS.

## Conclusion

This study demonstrated that CRF has greater association with the prevalence of MS compared with PA in Chinese midlife women. The potential benefits of attaining greater CRF should be promoted, particularly among those who are sedentary. However, no significant differences were observed in level of CRF between metabolically healthy but overweight/obese subjects and their metabolically abnormal and overweight/obese peers. The interrelationships between CRF, obesity, and MS needs further study.

## Competing interests

The authors declare that they have no competing interests.

## Authors’ contributions

RY contributed to the writing of manuscript, data collection and analysis. FY contributed to data collection and analyses. SCH developed the concept, planned data analyses and designed the study. JW developed the concept, planned data analyses, designed the study and writing of manuscript. We express our gratitude to our study subjects for their excellent collaboration. All authors read and approved the final manuscript.

## Pre-publication history

The pre-publication history for this paper can be accessed here:

http://www.biomedcentral.com/1471-2458/13/614/prepub
